# Should Clinical Ethicists Be Informed About Case Resolutions?

**DOI:** 10.1007/s10730-025-09549-6

**Published:** 2025-05-14

**Authors:** Marta Fadda

**Affiliations:** 1 Institute of Family Medicine, Università della Svizzera italiana (USI), Via Buffi 13, 6900 Lugano, Switzerland; 2 Clinical Ethics Commission (COMEC), Ente Ospedaliero Cantonale (EOC), Bellinzona, Switzerland; 3 Center for Bioethics, Harvard Medical School, Boston, USA

**Keywords:** Clinical ethics, Institutional ethics committees, Clinical ethicist, Case resolutions

## Abstract

The question of whether clinical ethicists should be informed of case resolutions remains unresolved. While the American Society for Bioethics and Humanities (ASBH) recommends retrospective case reviews to assess whether recommendations were followed, it frames this practice solely as a quality improvement measure. While quality enhancement is a compelling rationale for ensuring that clinical ethicists are informed of the resolutions of consultations, it is not the sole justification for such transparency. Access to case resolutions strengthens ethics education, enhances accountability and transparency, facilitates contributions to the field and advocacy, and mitigates the emotional uncertainty that can arise when ethicists lack closure on complex cases. Although concerns about confidentiality and administrative constraints must be considered, they should not hinder efforts to foster a more transparent consultation process.

## Introduction

The question of whether clinical ethics consultants should be informed about the actions or decisions taken following their consultation remains unclear, as there are no broad justifications, formal guidelines, or established procedures addressing this issue within the profession. Additionally, there is a lack of empirical data on current practices or outcomes regarding case follow-up. The American Society for Bioethics and Humanities (ASBH) describes the role of clinical ethicists as facilitating clearer thinking about the ethical implications of actions to support informed decision-making (American Society of Bioethics and Humanities, 2011). The ASBH describes the general goal of ethics consultation as enhancing the quality of health care through the identification, analysis, and resolution of ethical questions or concerns (American Society of Bioethics and Humanities, 2011). Additional objectives include identifying and analyzing value uncertainties or conflicts, facilitating conflict resolution, and promoting practices that align with ethical norms and standards, all with the goal of fostering an ethically supportable consensus (American Society of Bioethics and Humanities, 2011). As such, the primary focus of these guidelines remains on the consultation process rather than the resulting decisions or actions. However, in discussing how ethics consultation services should engage in quality assessment and improvement, one suggested approach by the ASBH is reviewing cases *post hoc* with the ethics committee to evaluate whether recommendations were followed and how they influenced clinical decision-making (American Society of Bioethics and Humanities, 2011)​. This supports the argument that clinical ethicists should be kept informed of case resolutions to refine their practice.

While quality enhancement is a compelling rationale for ensuring that clinical ethicists are informed of the resolutions of consultations, it should not be the sole justification. Beyond improving the quality of ethics consultation services, access to case resolutions serves several critical functions. It enhances ethics education and professional development by providing concrete examples for reflection and learning. It also strengthens accountability and transparency, reinforcing trust in the ethical decision-making process within healthcare institutions. Moreover, it fosters a more effective contribution to the field and advocacy for patients and healthcare professionals alike. Finally, ensuring ethicists are aware of case resolutions mitigates the emotional and moral uncertainty that can arise when they are left without closure in complex or high-stakes cases. Of course, such transparency must be balanced with legitimate concerns about confidentiality and administrative constraints. However, recognizing and managing these challenges should not preclude efforts to promote a more open and ethically engaged consultation process.

## Training and Professional Development

Clinical ethics is a dynamic discipline that evolves through reflection, case-based learning, and iterative improvement (Branch & George, 2017). The ASBH guidelines affirm that ethics education is a key responsibility of clinical ethics consultants (American Society of Bioethics and Humanities, 2011), and several clinical ethics committees have an institutional mandate to fulfill this role. Many committee members serve as educators in medical and nursing curricula and as ethics trainers in a range of healthcare settings. Real cases, particularly those drawn from institutional practice, offer richer, more context-specific learning compared to abstract cases from the literature, which often rely on historical cases from a limited number of countries. Integrating case resolutions into ethics education would enhance discussions and better prepare future clinical ethicists and healthcare professionals for the complexities of ethics decision-making in healthcare. At the same time, the ASBH guidelines underscore the need for ongoing professional development, urging institutions to support ethics consultants through continuing education and training (American Society of Bioethics and Humanities, 2011). Access to case resolutions is a critical component of this professional development, for it enables consultants to engage in reflective practice and assess the impact of their ethical interventions. By reviewing case resolutions, consultants can assess how their recommendations influenced clinical decision-making, identify areas for personal improvement, and refine their ethical reasoning over time.

### Accountability and Transparency

If clinical ethics consultants are expected to preserve professional integrity by refraining from endorsing practices they deem ethically problematic (American Society of Bioethics and Humanities, 2011), then being informed about case resolutions is essential. Without access to case resolutions, consultants cannot assess whether their ethical guidance has been misinterpreted, misused, or disregarded in ways that contradict agreed-upon ethical standards. Awareness of case resolutions allows clinical ethics consultants to ensure that their recommendations contribute to ethically sound decision-making rather than inadvertently legitimizing questionable practices. It also enables them to refine their approach and address potential misunderstandings (Shea, 2024). Furthermore, transparency in case follow-up reinforces the accountability of ethics consultation services, ensuring that ethical guidance is not treated as a mere formality but as a meaningful contribution to patient care and institutional decision-making. Without this information, clinical ethics consultants risk being complicit in ethical compromises they would otherwise challenge, undermining the very integrity they are meant to uphold.

## Contribution to the Field and Advocacy

Ethics consultations often surface systemic issues — such as gaps in institutional policies, disparities in patient care, or recurring ethical challenges– that warrant broader attention. However, because these cases often go unpublished or are addressed only in informal discussions, they fail to contribute to the broader evidence base needed to inform institutional policy and practice. Considering this gap, clinical ethicists may be uniquely positioned to drive meaningful change within healthcare institutions. Access to case resolutions enables ethics consultants to recognize recurring ethical concerns, transform anecdotal experiences into actionable data, and advocate for systemic change for both patients and healthcare providers (Kon, 2012). Some scholars emphasize the duty of ethical advocacy (Antommaria, 2012; Milliken, 2024), asserting that consultants should be able to raise institutional ethics issues without fear of retaliation (Rasmussen, 2012). Without insight into case resolutions, ethicists are constrained in their ability to track patterns, assess the impact of their recommendations, and drive meaningful reforms. By incorporating case resolutions into institutional learning, ethics consultants can move beyond addressing individual dilemmas to reinforce the ethical foundations of healthcare organizations and advancing broader societal change toward health justice.

## Reducing Emotional Uncertainty

Ethicists, much like clinicians, engage deeply with cases involving high-stakes decisions, such as end-of-life care, resource allocation, and informed consent disputes. When they are left unaware of how a case was ultimately resolved, research shows that they may experience emotional distress and professional dissatisfaction, particularly when their ethical reasoning suggests a sound course of action but they are left uncertain about its practical impact (Bosek & Fulmer, 2018). The absence of closure can lead to rumination and psychological burden, undermining professional well-being. Moreover, without insight into case resolutions, ethicists miss opportunities to evaluate whether their guidance was effective, realistic, and aligned with clinical constraints, raising questions about the value of their role. The Euro-MCD (European Moral Case Deliberation instrument) includes “concrete resolution” as a key outcome of clinical ethics support (Svantesson et al., 2014). This means that ethics consultations should not only facilitate reflection but also lead to clear, actionable decisions that can be implemented in clinical practice. Ensuring that clinical ethicists are informed of case resolutions aligns with this principle, as it allows them to assess whether their recommendations contributed to meaningful ethical decision-making and problem-solving.

## Managing Confidentiality and Administrative Concerns

Implementing a system for informing clinical ethicists about case resolutions raises important considerations regarding confidentiality and administrative feasibility. While ethicists are already bound by institutional confidentiality policies, concerns may arise regarding the extent and nature of the information shared. A key challenge is balancing transparency with the need to protect patient privacy and clinical discretion. One possible approach is to provide ethicists with case summaries that highlight whether ethical recommendations were followed, modified, or disregarded, without disclosing sensitive details. This would allow ethicists to assess the broader impact of their consultations while mitigating privacy concerns. From an administrative standpoint, a structured yet minimally burdensome process is essential. Clinicians may perceive follow-up requests as an additional workload, particularly in high-pressure clinical environments. To address this, case resolution updates could be integrated into existing documentation workflows or electronic health records, allowing for streamlined reporting. Alternatively, ethics committees could designate a liaison responsible for collecting and summarizing feedback at predefined intervals, such as 30 days post-consultation. Case resolutions could then be presented and discussed during regular committees meeting. Clear institutional guidelines should delineate the scope of information to be shared and establish the ethicist’s role in the post-consultation phase.

## Conclusion

Unlike clinicians, who remain involved in ongoing patient care, clinical ethicists typically function in a consultative capacity– offering ethical guidance but rarely being informed of case resolutions. In many institutions, there is no systematic practice of updating ethicists on how cases are ultimately resolved, even when their recommendations have played a significant role in the decision-making process. This absence of follow-up raises ethical, professional, and practical concerns. Without insight into the consequences of their consultations, ethicists are limited in their ability to refine their practice, contribute effectively to ethics education, foster transparency and accountability, and advocate for systemic improvements. Furthermore, the emotional and professional uncertainty that results from this lack of closure may diminish the meaningfulness of ethics consultation as a profession. Despite these concerns, there is little empirical data on whether informing ethicists of case resolutions is a routine practice. Future research should investigate current approaches to case follow-up in ethics consultation, identifying barriers and best practices across different institutions. Such an inquiry would be particularly relevant in settings like university hospitals, where professional ethicists could benefit from sharing experiences and strengthening networks. Moreover, ensuring that clinical ethics committees across institutions maintain consistent practices in ethics consultation—including access to case resolutions—would contribute to the broader professionalization of clinical ethics (Porz, 2020). Recognizing case follow-up as an essential component of ethical reflection and institutional learning would not only support the development of clinical ethics as a discipline but also reinforce its role in improving patient care.
